# The Future Is Now—Prospective Study of Radiosurgery for More Than 4 Brain Metastases to Start in 2018!

**DOI:** 10.3389/fonc.2018.00380

**Published:** 2018-09-13

**Authors:** David Roberge, Paul D. Brown, Anthony Whitton, Chris O'Callaghan, Anne Leis, Jeffrey Greenspoon, Grace Li Smith, Jennifer J. Hu, Alan Nichol, Chad Winch, Michael D. Chan

**Affiliations:** ^1^Department of Radiation Oncology, Centre Hospitalier de l'Université de Montréal, Montreal, QC, Canada; ^2^Department of Radiation Oncology, Mayo Clinic, Rochester, MN, United States; ^3^Division of Radiation Oncology, Juravinski Cancer Centre, Hamilton, ON, Canada; ^4^Canadian Cancer Trials Group, Kingston, ON, Canada; ^5^Community Health and Epidemiology, College of Medicine, University of Saskatchewan, Saskatoon, SK, Canada; ^6^Department of Radiation Oncology, MD Anderson Cancer Center, Houston, TX, United States; ^7^Department of Public Health Sciences, University of Miami School of Medicine, Miami, FL, United States; ^8^BC Cancer Agency, Vancouver Centre, Vancouver, BC, Canada; ^9^Department of Radiation Oncology, Wake Forest Baptist Medical Center, Winston-Salem, NC, United States

**Keywords:** brain metastasis, clinical trials, Phase III as topic, whole brain radiation therapy, radiosurgery, neurocognition

## Abstract

Stereotactic radiosurgery (SRS) has replaced whole brain radiotherapy (WBRT) as standard therapy for most patients with four or fewer brain metastases due to improved cognitive outcomes and more favorable health related quality of life (QoL). Whether SRS or WBRT is the optimal radiation modality for patients with five to fifteen brain metastases remains an open question. Efforts are underway to develop prospective evidence to answer this question. One of the planned trials is a Canadian Cancer Trials Group (CCTG)-lead North American intergroup trial. In general cancer treatments must have two basic aims: prolonging and improving QoL. In this vein, the selection of overall survival and QoL metrics as outcomes appear obvious. Potential secondary outcomes are numerous: patient/disease related, treatment related, economic, translational, imaging, and dosimetric. In designing a trial, one must also ponder what is standard WBRT—specifically, whether it should be associated with memantine. With the rapid accrual of an intergroup trial of hippocampal-sparing WBRT, we may find that the standard WBRT regimen changes in the course of planned trials. As up-front radiosurgery is increasingly used for more than 4 brain metastases without high level evidence, we have a window of opportunity to develop high quality evidence which will help guide our future clinical and policy decisions.

## Background

The development of brain metastases is an unfortunate and common complication in oncology and can occur in 10–30% of cancer patients and up to half of patients with metastatic disease (1). The traditional treatment for many patients with brain metastases has been whole brain radiotherapy (WBRT), although stereotactic radiosurgery (SRS) has replaced WBRT as the standard therapy for most patients with four or fewer brain metastases due to more favorable cognitive and quality of life (QoL) outcomes (Table 1) (4, 7).

**Table 1:** Published studies of radiosurgery for < 4 brain metastases.

**Study**	**Treatment**	**Local control (1 yr) (%)**	**Distant failure (1 yr) (%)**	**Overall survival (1 yr) (%)**
RTOG 95-08 (2)	WBRT	71	33	23
	WBRT + SRS	82	27	29
EORTC 22952 (3)	SRS	70	44	47
	SRS + WBRT	87	28	46
MDACC (4)	SRS	67	55	60
	SRS + WBRT	100	27	21
JROSG-99-1 (5)	SRS	76	63	28
	SRS + WBRT	90	42	39
Alliance N0574 (6)	SRS	73	30	39
	SRS + WBRT	90	8	36

Although radiosurgery has historically been technically complex for patients with numerous metastases, each of the treatment delivery devices (Gamma Knife, Cyberknife and the isocentric linac) now permits more rapid treatment of multiple metastases. Although patients with more than 4 metastases are at greater risk of rapid distant brain failure we now know that number of brain metastases is not a reliable predictor of future intracranial progression. A multi-institutional nomogram was developed to predict the development of new brain metastases after primary SRS (8). A major finding from this study was that the nomogram was superior in predicting the development of new metastases in comparison to simply using the number of metastases (8).

Barriers to the adoption of radiosurgery for multiple metastases have been decreasing and we know from retrospective and prospective research that SRS alone is feasible in patients with up to 10 brain metastases (9). Additionally, radiosurgery for as many as 15 brain metastases has been found to be safe, notably in a series of more than 300 patients (10).

The question of whether SRS or WBRT is the optimal modality in patients with five to fifteen brain metastases is significant from a societal and medical resources standpoint. In the United States, the charges related to SRS can be considerably higher than those of WBRT (1, 11). An analysis of 2008 non-Medicare charges in different geographic regions of the United States found WBRT charges ranged from $9,201 to $17,003 while SRS charges ranged from $40,715 to $65,000. Methodologies for financing radiotherapy vary across Canada but the marginal cost to the state insurer of a single SRS or WBRT course, including physician billing and memantine, can be similar and as low as $3500–4000 (USD). Quantifying therapy-associated costs can be particularly complex in patients with multiple brain metastases, as such patients are likely to undergo salvage procedures for new brain metastases. Therefore, the costs of salvage treatment need to be incorporated into economic comparisons.

It is important to develop high quality prospective evidence as adoption of SRS increases for patients with more than 4 metastases. Currently clinicians face ongoing uncertainties about the true cost burden of SRS vs. WBRT from payer and provider perspectives, as well as uncertainties about the comparative risk/benefit of these strategies for survival, CNS control, QoL, and neurocognitive function in patients with more than four metastases. Published reports have already suggested value of SRS in improving cost utility in the population of 1–4 brain metastases. Lal et al. reported a cost-effectiveness analysis of a randomized trial of SRS vs. SRS + WBRT for 1–3 brain metastases and found that SRS alone had a higher average cost but was associated with an improvement in QALYs with an incremental cost-effectiveness ratio of $41,783 per QALY (12). Savitz et al. performed a cost-effectiveness analysis using a Markov model and found that SRS was a cost-effective treatment option, even in patients who had prognoses of six months or less (13). Accordingly, it is important that SRS for five to fifteen brain metastases is studied in a prospective multi-institutional cooperative group trial to evaluate cost, as well as cost-effectiveness and cost utility.

**Figure 1:**
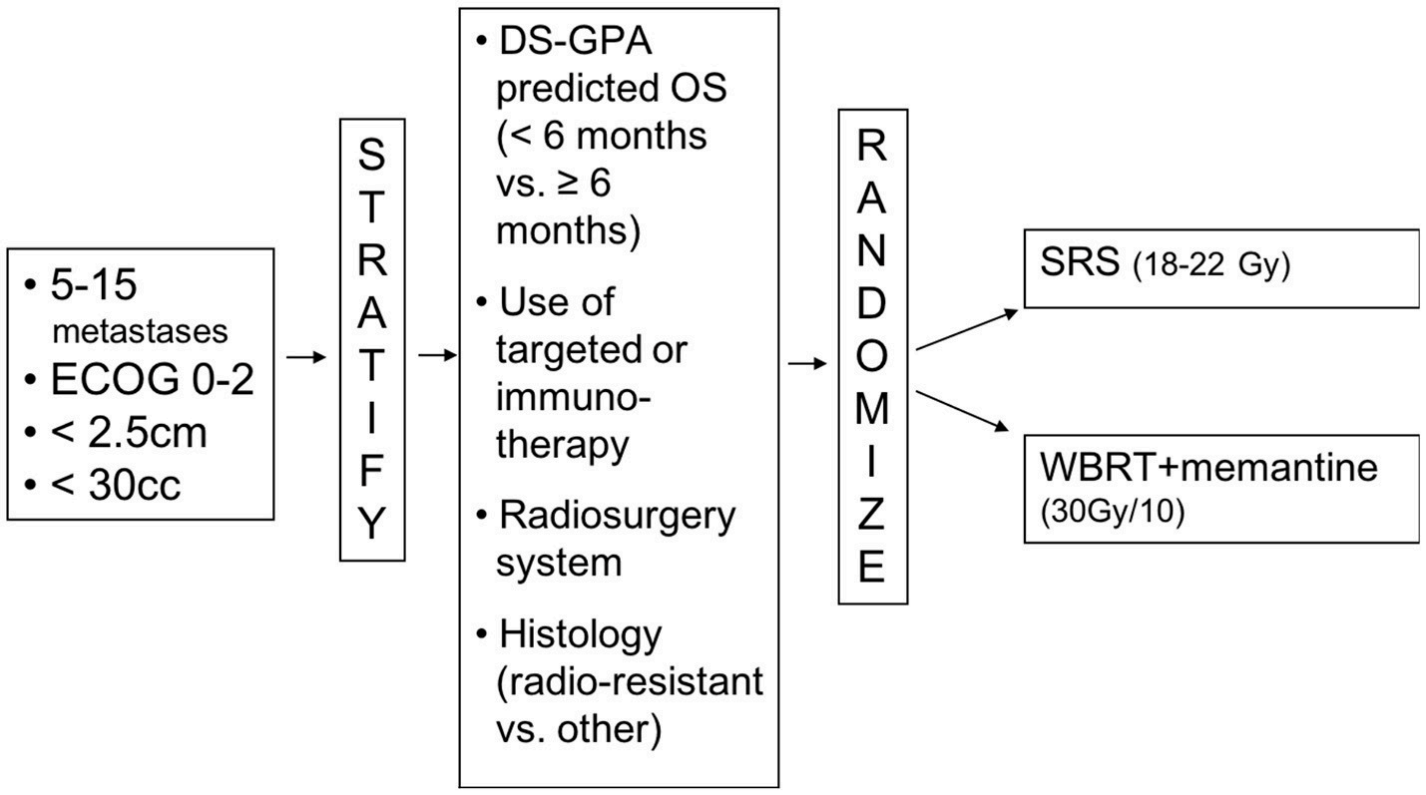
CCTG CE.7 Study Schema.

Having made a strong contribution to an intergroup trial, N107C/CEC.3, comparing SRS to WBRT following surgical resection of brain metastases, the Canadian Cancer Trials Group (CCTG) was keen to lead a trial for brain metastases. The concept of a trial of WBRT vs. radiosurgery for brain metastases was first presented to the CCTG's CNS group in April 2016. Over the following year, the members were surveyed and the trial concept was refined. Dr Chan, as part of the Alliance cooperative group, was recruited as co-principal investigator and the trial was submitted to NCI Cancer Therapy Evaluation Program (CTEP). After minor revisions, the trial was approved by CTEP in January 2018. The central IRB approval followed in March 2018 (Figure 1). The trial is thus on track to open to accrual across North America in the summer of 2018.

This chapter aims to review the background of the trial and the choices that had to be made in its design. We hope to garner enthusiasm for this important trial. We also hope to illustrate the important role of intergroup trials as we review the landscape of Phase III research within which our trial will fit. The difficulty in completing such trials is exemplified by the recent decision of Dr Zindler and his Dutch co-investigators to end a trial planned to recruit 230 patients with 4–10 metastases (NCT02353000). The primary endpoint of the trial had been QoL—specifically variation in EQ-5D-5L. Accrual ended after 2 years with 30 patients randomized to hypofractionated WBRT (without memantine) or SRS (14).

### Selecting outcomes

Cancer treatments should have two basic aims: prolonging and improving QoL. In this vein, the selection of overall survival and QoL metrics (neurocognitive) as co-primary outcomes for an intergroup trial were obvious.

As one would expect that intracranial control will be better with WBRT than SRS, how could SRS improve overall survival? In addition to its direct toxic effects, WBRT likely delays initiation or re-initiation of increasingly active systemic therapy. Patients initially treated with WBRT will have inferior local control of their metastases and may have less aggressive subsequent management of their intracranial disease. Radiosurgery may thus provide a small survival benefit over WBRT.

The rationale for improvements in QoL is more straightforward. Although quality of life can be defined as a state of general wellbeing reflecting physical, psychological, and social wellbeing, the aspects of QoL that are most likely to be affected in this study are treatment related symptoms and overall QoL. To evaluate QoL the EORTC core questionnaire QLQ-C30, in conjunction with the brain module QLQ-BN20, were selected. Patient performance status, and the EQ-5D questionnaire will also be used.

The EORTC QOL questionnaire core-30 (QLQ-C30, version 3); and the EORTC QOL questionnaire—brain module (QLQ-BN20) have robust psychometric properties and are highly consistent across different language-cultural groups. The EORTC QLQ-C30 consists of 30 questions which comprise five function scales: physical, role (interference of disease with family life or social activities), emotional, cognitive, and social; six single-item scales including dyspnea, insomnia, appetite loss, constipation, diarrhea, and financial effect of tumor and treatment; and overall QOL. EORTC QLQ-BN20 is designed for use with patients with brain tumors and has 20 items that assesses visual disorders, motor dysfunction, communication deficit, various disease symptoms (e.g., headaches and seizures), toxic effects of treatment, and future uncertainty. Since mood disturbances may influence cognitive function, it will be important to interpret QOL data in light of neurocognitive test results.

The neurocognitive evaluations to be used in this study were chosen on the basis of accepted standardization and psychometric principles, published normative data, relevance to general neurocognitive status, and brevity of battery. The tasks selected have either low practice effect or include multiple equivalent formats. Similar variations of this battery have been utilized in multiple multi-institutional trials including N107C/CEC.3, N0574, N0577, E3F05 and RTOG 0614 (15, 16). The tests include:

- Memory (5 min): Hopkins Verbal Learning Test (HVLT) (17).
- Fluency (5 min): Controlled Oral Word Association Test (COWAT) (18).
- Visuomotor speed and attention: Trail Making Test A (3 min) (19).
- Executive function: Trail Making Test B (5 min) (19).
- Delayed Memory (5 min): Recall and Recognition of Word List encoded from the HVLT (20).

### What is standard of care?

In designing a Phase III trial of radiosurgery for patients with more than 4 brain metastases, one has to determine the standard treatment for these patients. Reflexively, one would presume that WBRT would be considered the standard. Although WBRT is commonly used, what is the evidence that it improves either overall survival or QoL? A report from Horton and colleagues from the Eastern Cooperative Oncology Group described a study of 48 patients randomized in a three-arm trial to a combination of steroids and whole-brain radiotherapy (21). There was no report of QoL and the overall survival was 14 weeks in the arms containing radiotherapy and 10 weeks with prednisone alone—no test of statistical significance was performed. Recognizing the weakness of this evidence, a larger trial was designed to randomize patients with NSCLC to WBRT vs. supportive care. In this QUARTZ trial overall survival was nearly identical in both arms (9.2 weeks with WBRT and 8.5 weeks without) and there was no significant difference in patient reported QoL (22). In melanoma, patients have been randomized to chemotherapy (the relatively ineffective drug fotemustine) with or without WBRT (23). Although there was an improvement in progression free survival, the overall survival curves were indiscernible.

Thus, for many patients with less favorable prognoses, no radiation treatment has been clearly demonstrated to be better than supportive care. At the other end of the brain metastasis spectrum, patients with targetable mutations are increasingly being offered systemic therapy. As a recent example, the FLAURA trial for first line treatment of patients with advanced EGFR-mutated NSCLC included patients with previously untreated brain metastases (24). One hundred and sixteen patients with brain metastases were randomized to osimertinib or a choice of gefitinib or erlotinib. Survival data is immature but osimertinib offers a better disease response with less toxicity. One could argue that current evidence supports the use of a TKI alone for patients with asymptomatic brain metastases and an exon 19 deletion or L858R mutation.

It becomes rapidly clear that an evidence-based standard of care is elusive and complex. The question to be answered may then better be expressed as: “in those patients treated with radiation for more than 4 metastases, what is an accepted standard of care against which to compare radiosurgery.” The answer to this question is thus WBRT. What is left is to choose details of the WBRT regimen. Although no WBRT fractionation has shown advantage in survival or cognitive function, it was sensible to select 30Gy in 10 fractions as a regimen acceptable in North America.

Memantine is an N-methyl-D-aspartate (NMDA) receptor antagonist studied in a placebo-controlled, double-blind, randomized trial in patients with brain metastases receiving WBRT (RTOG 0614) (15). Patients received WBRT and were randomized to receive placebo or memantine during and after WBRT for a total of 24 weeks (10 mg twice a day). Between 2008 and 2010, 554 patients were accrued. Grade 3 or 4 toxicities and study compliance were similar between arms. Although the difference in the primary endpoint (decline in HVLT-R at 24 weeks) did not quite reach statistical significance (*p* = 0.059), this may be attributable to the fact that there were fewer analyzable patients than expected significantly impacting study power. Patients in the memantine arm did however have a significantly longer time to cognitive decline (HR 0.78; 95% CI, 0.62–0.99; *p* = 0.02). Following publication of this data, controversy remains but memantine has been integrated into the standard care of patients treated with WBRT in selected practices in the United States and Canada.

Buoyed by favorable Phase II results of hippocampal sparing, Drs Brown, Gondi and co-investigators at the NRG initiated a Phase III trial of hippocampal avoidance in the management of brain metastases (25). After having accrued briskly, the protocol closed in March 2018 and preliminary results are expected later in 2018. Should the results suggest a cognitive benefit to hippocampal sparing, this will replace WBRT for those patients in whom the location of the metastases permits hippocampal sparing (to be eligible for the trial, patients could not have metastases within 5 mm of either hippocampus). Although not tested in CC001, unilateral sparing (especially in the dominant hemisphere) could also be used in those patients with unilateral encroachment on the hippocampal avoidance region—especially when the dominant hemisphere can be spared.

### Statistics

As the CCTG trial has co-primary endpoints (overall and neurocognitive progression-free survival), these endpoints are planned to be analyzed jointly. The interpretation is pre-specified:

- If SRS is found to be superior for neurocognitive progression-free survival and non-inferior for overall survival, the study would establish SRS as the standard of care for patients with 5–15 metastases.
- If SRS is found to be superior in terms of neurocognitive progression-free survival but slightly worse in terms of overall survival, then SRS may still have clinical use. In this situation, secondary endpoints, including QoL and economic endpoints may be of particular interest in making policy and treatment decisions.
- If SRS is neither superior in terms of cognition or overall survival, WBRT would be clarified as the standard of care for patients with more than 4 metastases.

In calculating sample size, it was assumed that neurocognitive progression would occur in 50% for patients undergoing WBRT by 6 months post-treatment. The estimated median overall survival in the WBRT was estimated to be 7.5 months. Based on these assumptions, the trial was designed to have a 90% power to detect a 40% risk reduction in the risk of the neurocognitive progression using a 5% 2-sided test. With the sample size of 206 patients, the power to detect a 15% reduction in the hazard of death in the SRS arm would thus be 80%. Based on prior experience with brain metastases in the Cancer Trials Support Unit (CTSU) network, this sample size appears to be reasonable for a trial accruing over 3 years.

### Landscape

The question of the best radiotherapeutic management of multiple brain metastases is one which solicits much interest. The CCTG/Intergroup initiative should be the trial which rallies the most institutions but there are other ongoing initiatives of interest:

- Building on prior success in brain metastases trials, the group at MD Anderson are currently performing a trial of radiosurgery vs. WBRT for patients with 4–15 metastases (NCT01592968). The primary outcomes are cognitive decline at 4 months (as measured by change in HVLT-R) and local control. The control arm is WBRT 30Gy in 10 without memantine. The estimated trial accrual is 100 patients.
- In Boston, the groups at Brigham and Women's Hospital and the Dana Farber Cancer Institute plan to accrue 196 patients to a Phase III trial of radiosurgery (which can be fractionated) vs. WBRT for 5–20 metastases (NCT03075072). In this trial the comparator arm is WBRT with possible hippocampal sparing. The primary endpoint is QoL as measured by the MD Anderson Symptom Inventory—Brain Tumor (MDASI-BT).

Recognizing that there is a need to prevent the occurrence of new brain metastases while minimizing toxicity, alternatives to WBRT are of interest as adjuncts to radiosurgery. The most prominent endeavor is “METIS,” a trial which aims to improve intracranial control in patients with non-small cell lung cancer and up to 10 metastases (NCT02831959). Patients are randomized to radiosurgery with or without electrical fields—so called “tumor treating fields.” In 8 countries, 270 patients are to be randomized with time to intracranial progression as the primary endpoint.

Completing trials where one arm has WBRT can be a challenge, this is exemplified by the failure of the North American Gamma Knife Consortium to accrue in their trial of radiosurgery vs. WBRT for patients with 5 or more brain metastases (NCT01731704).

## Conclusion

Uncertainty remains as to the best approach to patients with more than 4 brain metastases which are amenable to radiosurgery. Current efforts, including an intergroup trial lead by the CCTG, should generate high quality evidence to inform clinical practice. Despite this new upcoming trial, clinical ambiguity may persist because of the high heterogeneous patient population and further translational research will be needed to better combine available treatments, identify biomarkers, and develop innovative approaches to metastases within the CNS.

## Author contributions

DR, PB, AW, CO, AL, JG, GS, JH, AN, CW, and MC participated in the design of the work and review of the manuscript. DR, CW, and MC performed much of the writing of the manuscript.

### Conflict of interest statement

DR: Elekta (research support), VMS, Accuray, Siemens (honoraria and research support), Brainlab, Pfizer, EMD Serono (honoraria). PB: Novella Clinical (DSMB), UpToDate (Contributor). AN: VMS (research support). The remaining authors declare that the research was conducted in the absence of any commercial or financial relationships that could be construed as a potential conflict of interest.
